# Supplementary material for: Targeting the Epigenetic Non-Coding RNA MALAT1/Wnt Signaling Axis as a Therapeutic Approach to Suppress Stemness and Metastasis in Hepatocellular Carcinoma
Source: Cells. 2020 Apr 20;9(4):1020. doi: 10.3390/cells9041020 (PMC7226390; doi:10.3390/cells9041020)
Supplement: Supplementary file 1 [file cells-09-01020-s001.pdf]

SUPPLEMENTARY INFORMATION

Targeting Epigenetics non-coding RNA MALAT1/Wnt signaling axis as a therapeutic approach to suppress stemness and metastasis in hepatocellular carcinoma

Hang-Lung Chang [1,2†], Oluwaseun Adebayo Bamodu [3,4], Jiann-Ruey Ong[5,6†], Wei-Hwa Lee[7], Chi-Tai Yeh[3,4,8] and Jo-Ting Tsai[9,10,11*]

1. Department of General Surgery, En Chu Kong Hospital, New Taipei City, Taiwan;
2. Department of Health Care Management, Yuanpei University of Medical Technology, Hsinchu, Taiwan;
3. Department of Hematology and Oncology, Cancer Center, Taipei Medical University - Shuang Ho Hospital, New Taipei City, Taiwan;
4. Department of Medical Research and Education, Taipei Medical University - Shuang Ho Hospital, New Taipei City, Taiwan;
5. Department of Emergency Medicine, School of Medicine, Taipei Medical University, Taipei.
6. Department of Emergency Medicine, Shuang-Ho Hospital-Taipei Medical University, New Taipei City.
7. Department of Pathology, Taipei Medical University-Shuang Ho Hospital, New Taipei City, Taiwan.
8. Department of Medical Laboratory Science and Biotechnology, Yuanpei University of Medical Technology, Hsinchu, Taiwan;
9. Department of Radiology, School of Medicine, College of Medicine, Taipei Medical University, Taipei, Taiwan;
10. Department of Radiology, Taipei Medical University - Shuang Ho Hospital, New Taipei City, Taiwan.
11. Graduate Institute of Clinical Medicine, College of Medicine, Taipei Medical University, Taipei City, Taiwan.

† Contributed equally to this work

*Correspondence: Jo-Ting Tsai, e-mail: 10576@s.tmu.edu.tw; Tel: +886-(2)-2490088 (ext. 8885); Fax: +886-2-2248-0900

Supplementary Table S1. Western blot antibodies sheet.

No.	Target	Dilution	Catalog	kDa
**1**	CD133	1:1000	#MAB4310	120
**2**	ALDH1	1:1000	ALDH1A1 (D4R9V) Rabbit mAb #12035	55
**3**	β-catenin	1:1000	β-Catenin (6B3) Rabbit mAb #9582P	92
**4**	GAPDH	1:1000	GAPDH Antibody (0411) (sc-47724)	37
**5**	P-Stat3	1:1000	Phospho-Stat3 (Tyr705) (D3A7) XP® Rabbit mAb	79,86
**6**	Stat3	1:1000	Stat3 (79D7) Rabbit mAb #4904	79,86
**7**	c-Myc	1:500	c-Myc Antibody (9E10) (sc-40)	55
**8**	CK19	1:1000	CK19 monoclonal antibody SAB3300019	43.9
**9**	Vimentin	1:1000	Anti-Vimentin antibody (ab137321)	57
**10**	Twist1	1:1000	Twist (Twist2C1a) Antibody sc-81417	28
**11**	Cyclin D1	1:1000	Cyclin D1 Antibody (A-12) (sc-8396)	38
**12**	Axin2	1:1000	Axin2 Antibody SAB3500619	93
**13**	LEF1	1:1000	LEF1 (C12A5) Rabbit mAb #2230	25~58
**14**	DKK1	1:1000	DKK1 Antibody #4687	30,35
**15**	β-actin	1:10000	β-Actin (8H10D10) Mouse mAb #3700	42

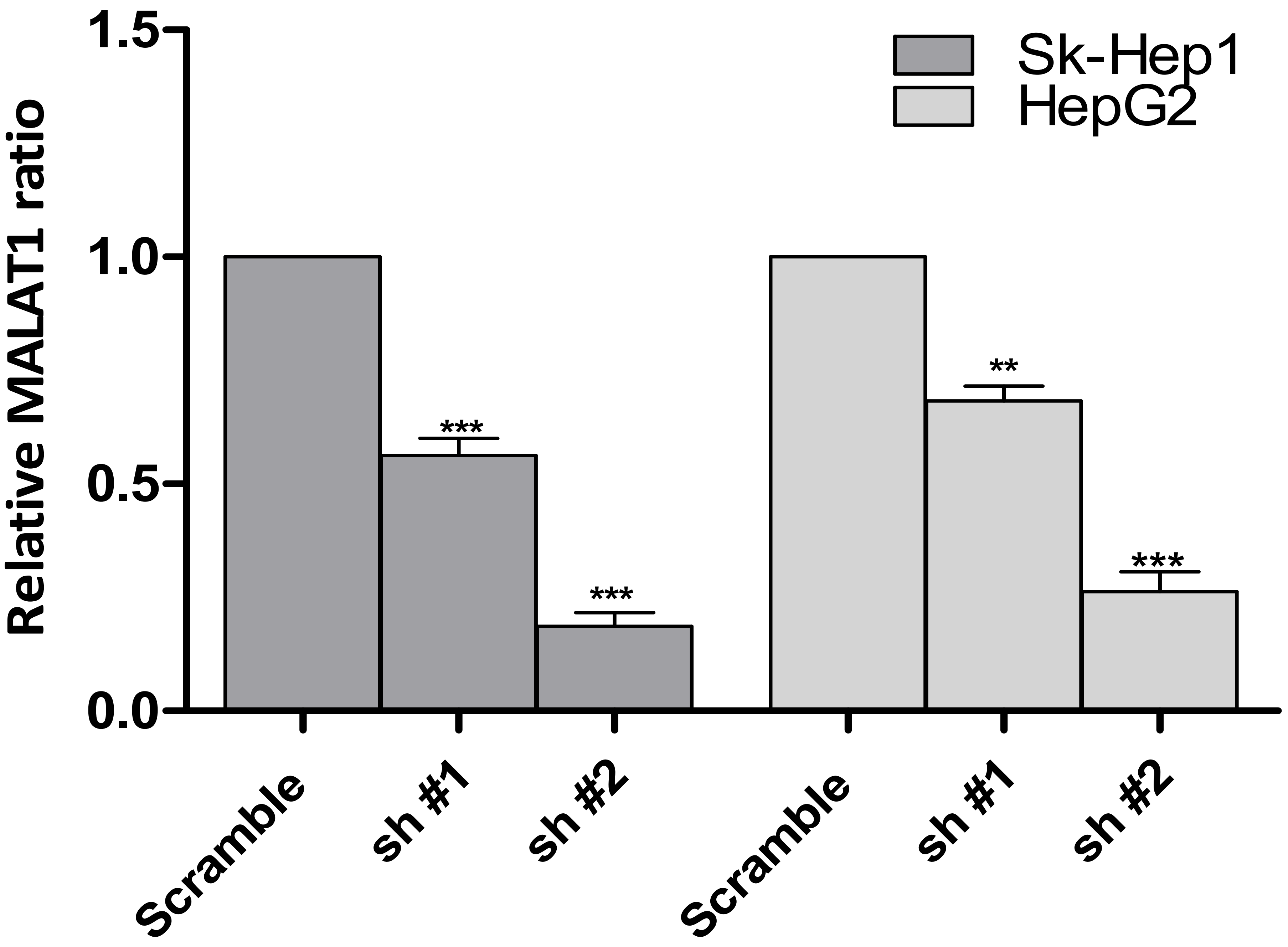

Supplementary Figure S1

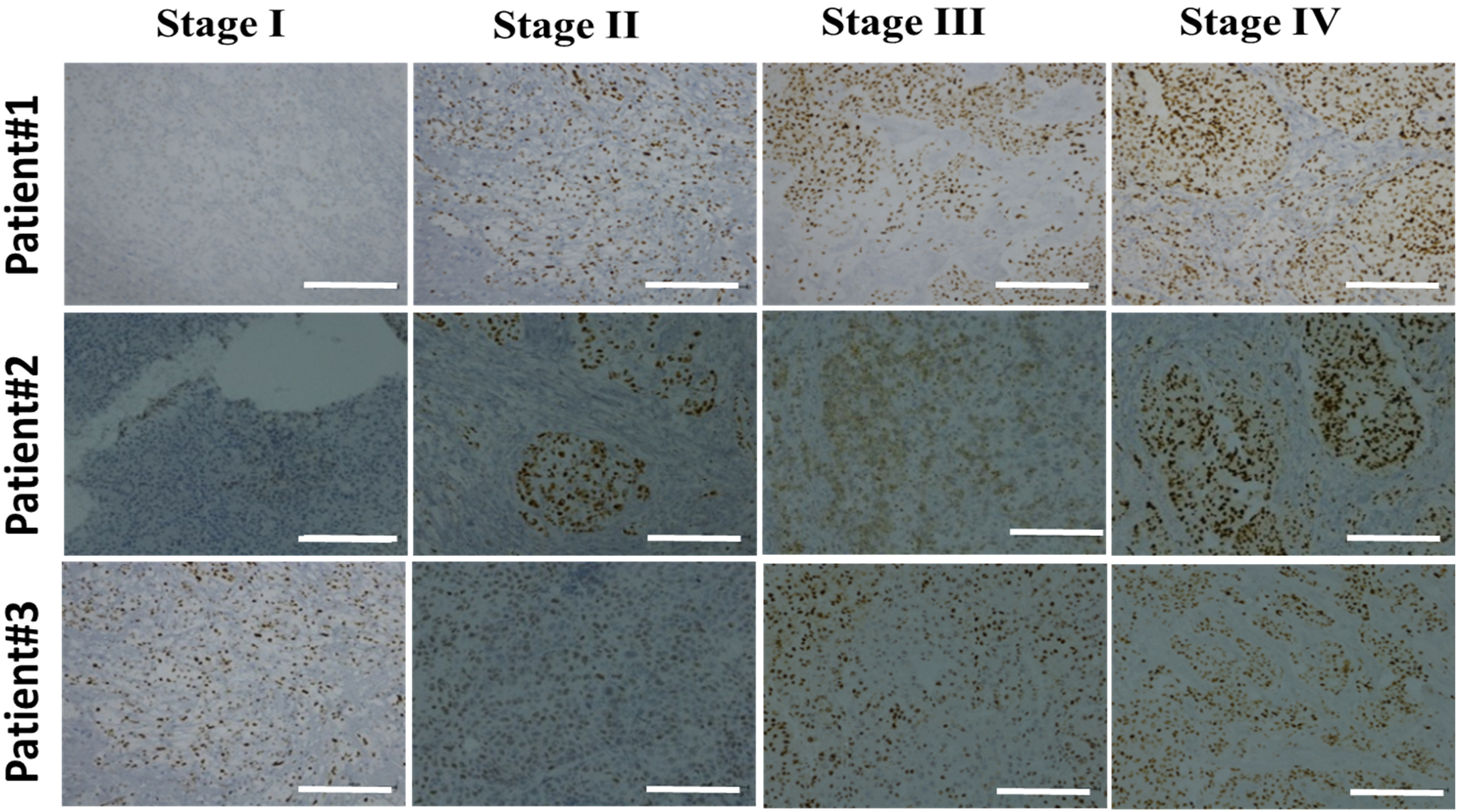

Supplementary Figure S2

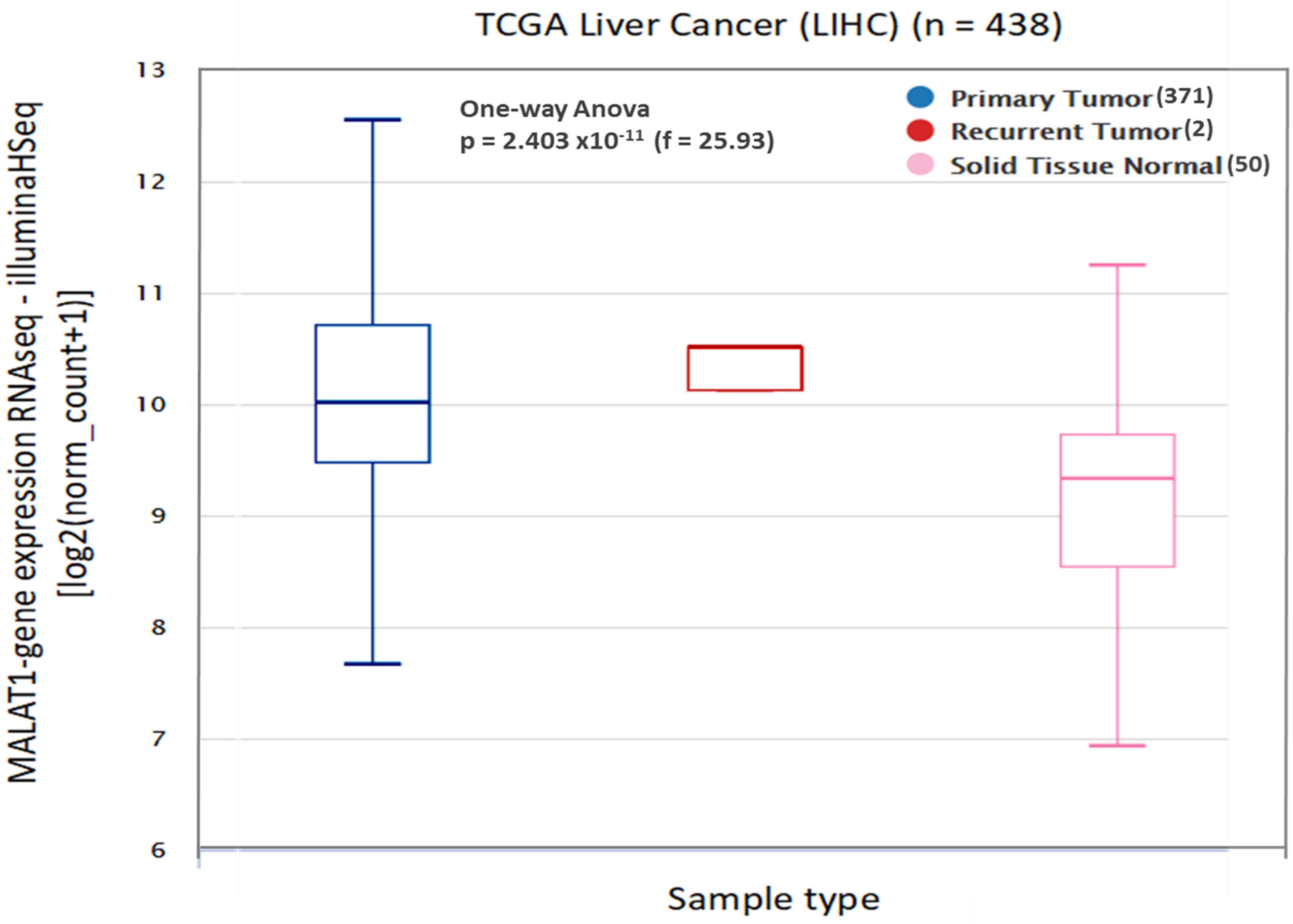

Supplementary Figure S3

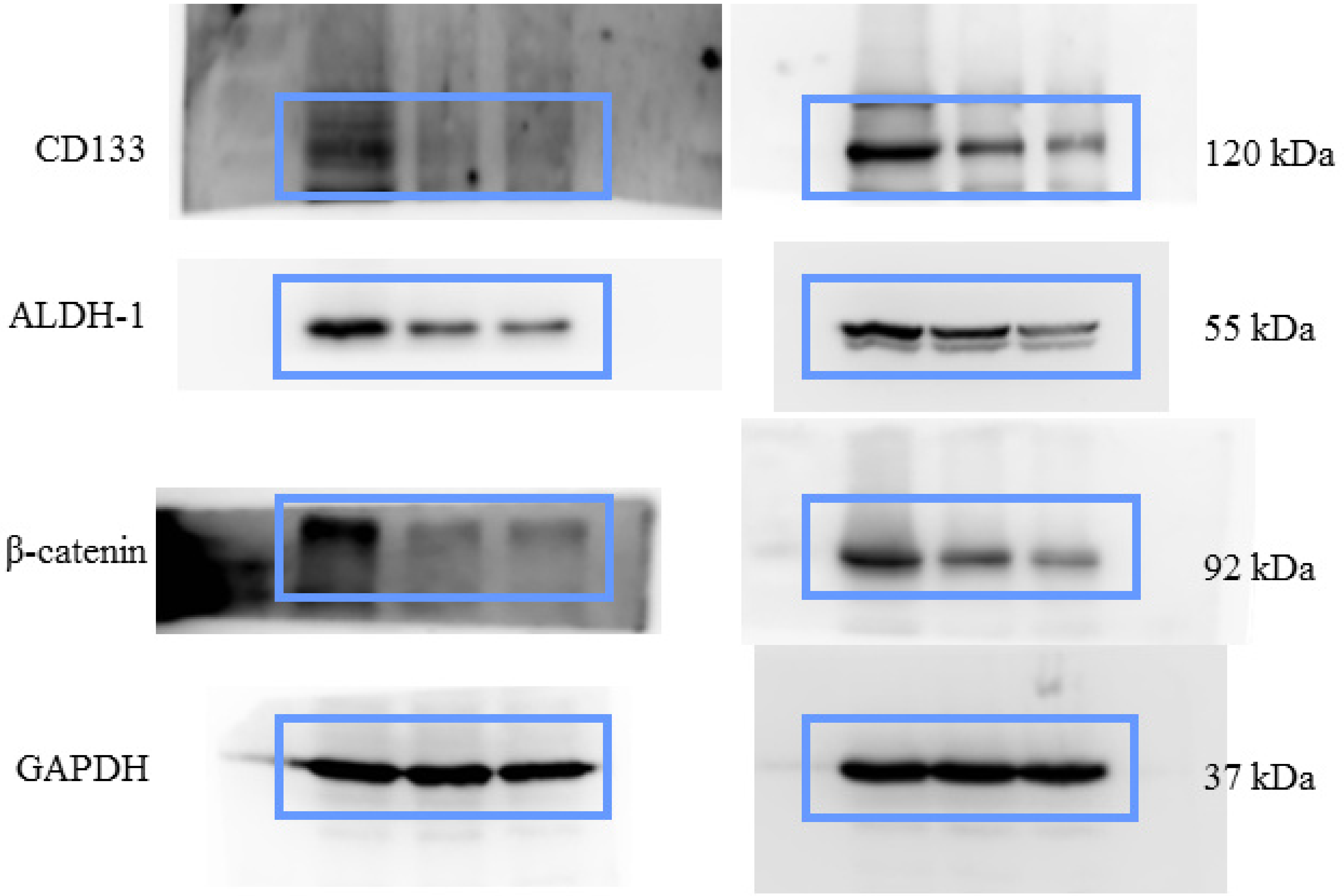

Supplementary Figure S4. Full-size blots of Figure 3B

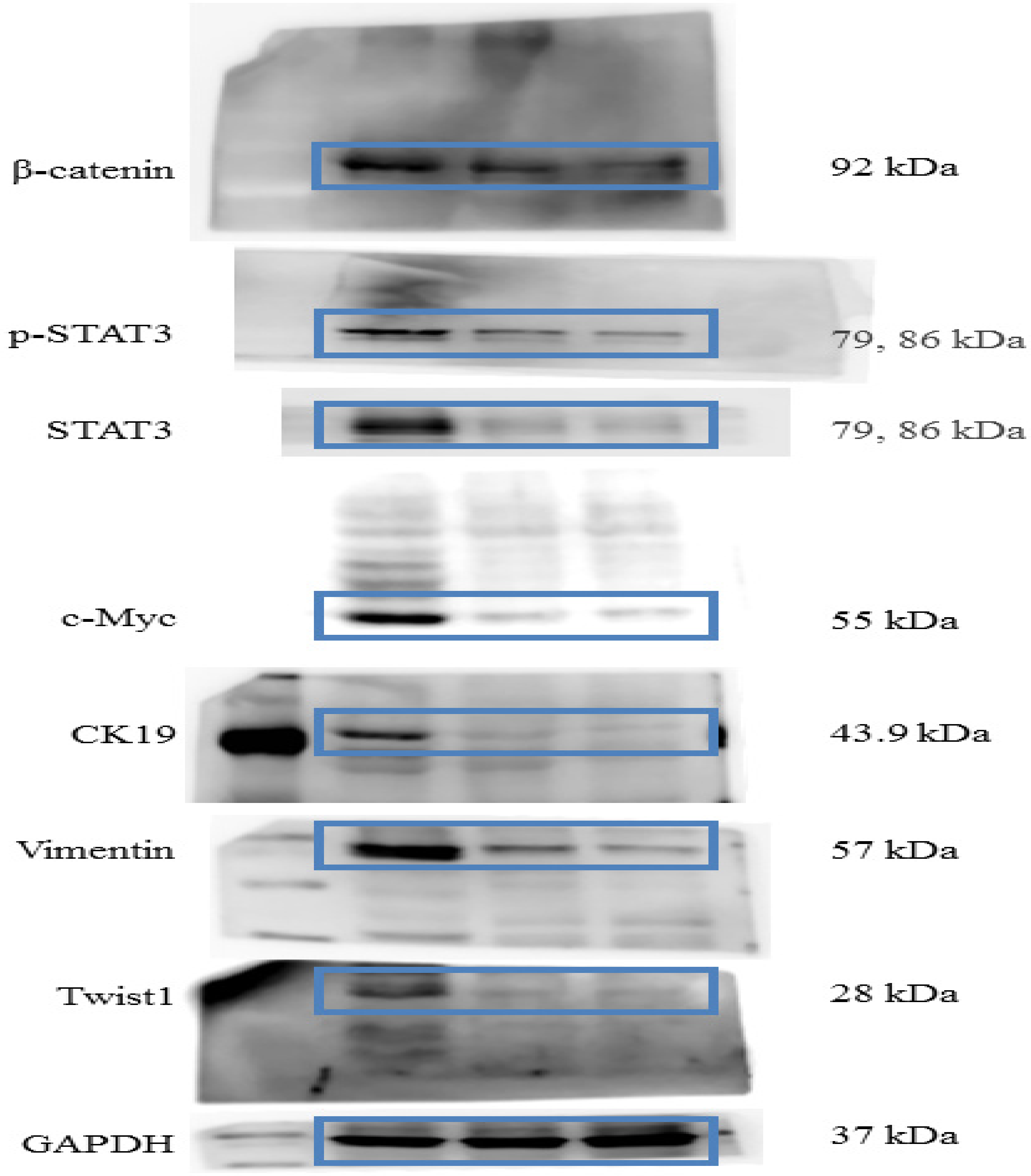

Supplementary Figure S5. Full-size blots of Figure 3D

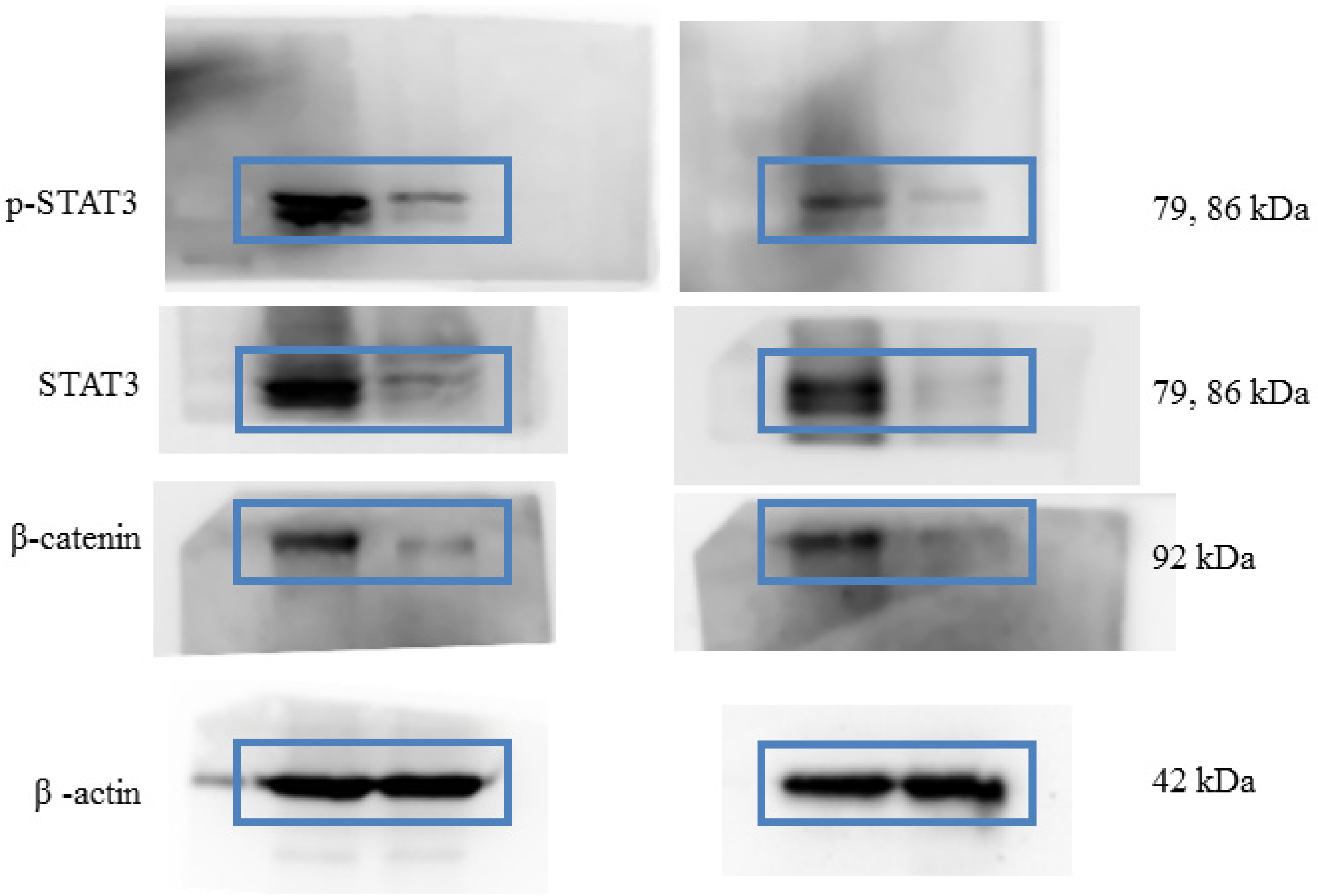

Supplementary Figure S6. Full-size blots of Figure 4C

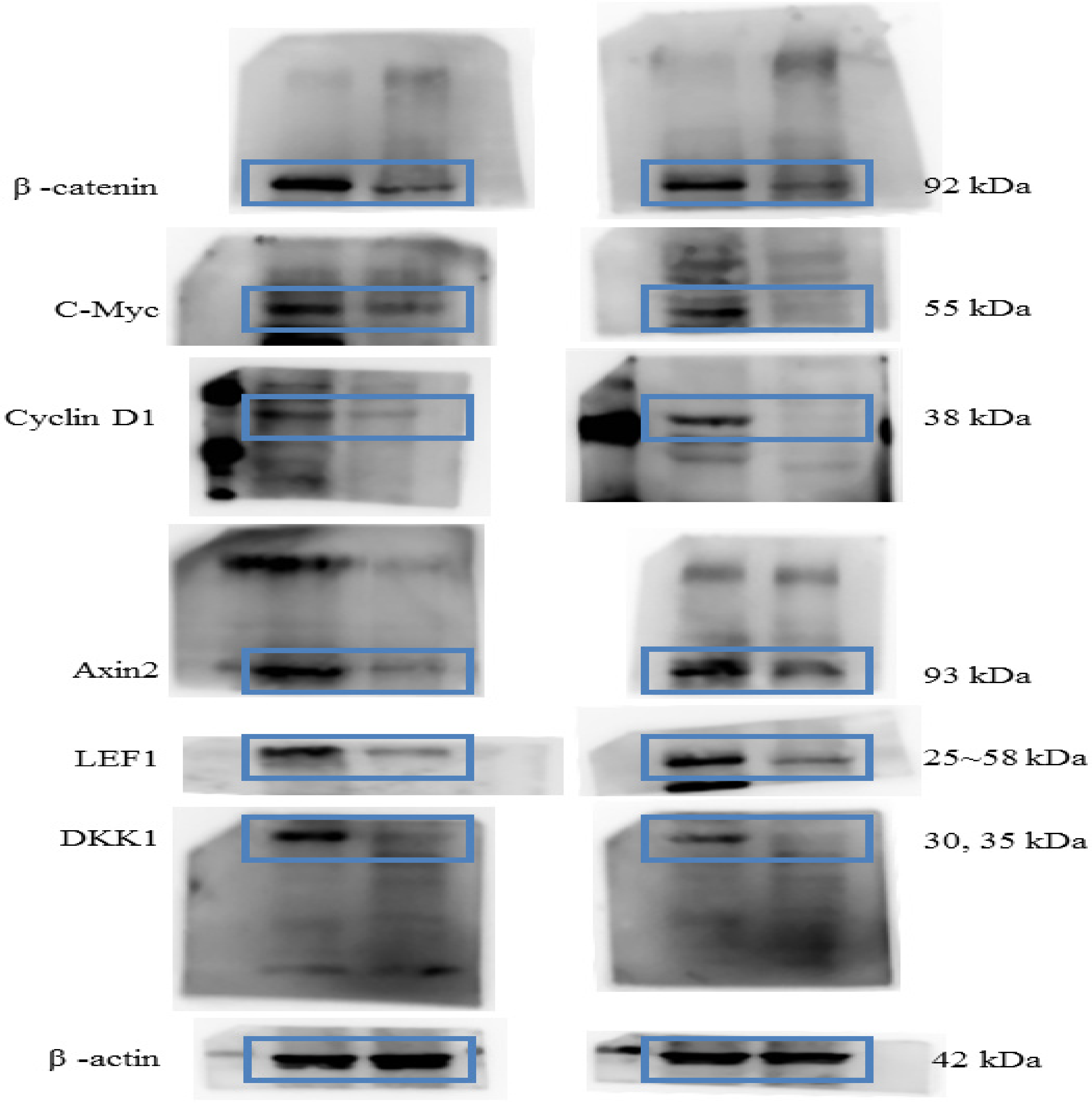

Supplementary Figure S7. Full-size blots of Figure 4D
